# Substitution of tyrosine with electron-deficient aromatic amino acids improves Ac-PHF6 self-assembly and hydrogelation†

**DOI:** 10.1039/d5ra03251b

**Published:** 2025-06-30

**Authors:** Shubhangini Singh Verma, Nitin Chaudhary

**Affiliations:** a a, Department of Biosciences and Bioengineering, Indian Institute of Technology Guwahati Guwahati 781 039 India chaudhary@iitg.ac.in +91-361-2582249 +91-361-2582224

## Abstract

The hexapeptide PHF6 (VQIVYK), an amyloidogenic peptide stretch from human tau, self-assembles *via* parallel in-register β-sheet formation, wherein Tyr residues are involved in aromatic stacking interactions. Ac-PHF6 (CH_3_CO-VQIVYK-NH_2_) forms a viscous solution in water but causes instant gelation of PBS and cell culture media. Aromatic substitutions have been reported in the literature to modulate the self-assembly of peptides. In this study, we perturbed the electronic properties of the sole aromatic residue in Ac-PHF6 and studied hydrogelation. The Tyr residue was substituted with Phe, and the phenyl moiety was then substituted with various electron-withdrawing groups at the *para* position. All peptides caused PBS gelation with comparable rheological properties. The structures underlying the hydrogels were β-sheet fibrils. The electron-deficient aromatic moieties improved self-assembly and hydrogelation. Ac-PHF6 and no other aromatic analog except the one having *p*-(trifluoromethyl)phenylalanine caused the gelation of deionized water. Water gelation caused by the *p*-(trifluoromethyl)phenylalanine-containing analog is likely hydrophobicity-driven.

## Abstract 2

Ac-PHF6 (CH_3_CO-VQIVYK-NH_2_) and its analogs with electron-deficient aromatic rings form hydrogels with comparable rheological properties.

## Graphical abstract

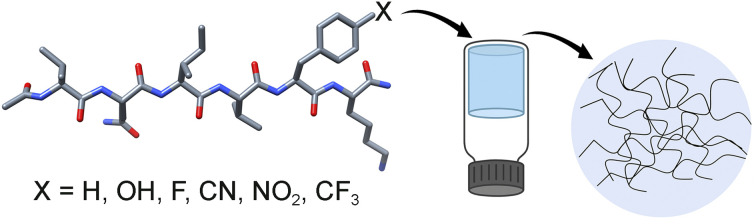

## Introduction

Hydrogels have emerged as promising soft materials in recent years. Their applications span very diverse areas.^1–6^ Peptides constitute an important class of self-assembling polymers.^7–12^ Hydrogels formed by self-assembling peptides have emerged as promising biomaterials.^13–32^ Hydrogels formed by native peptides offer significant advantages as they circumvent concerns related to toxicity and immunogenicity. Most peptide hydrogelators reported in the literature self-assemble to form β-sheet-rich fibrils. Such fibrils are long known to be associated with amyloid diseases. Amyloid fibrils, therefore, were considered pathogenic structures. As many functional amyloids have been identified in the past few decades, amyloid fibrils are no longer considered pathogenic fibrils.^33,34^ Amyloidogenic peptides, therefore, have gathered significant attention as biocompatible materials.

Amyloid peptides self-assemble *via* cross-β-sheet architecture to form fibrillar structures. Under appropriate conditions, such fibrillar structures can form robust three-dimensional hydrogels.^35,36^ Hauser and coworkers demonstrated hydrogel formation by short, *N*-acetylated amyloidogenic peptides.^37^ Banerjee and coworkers reported thermoreversible, pH-sensitive hydrogel formed by a human amylin tetrapeptide, hIAPP_24–27_.^17^ Maji and coworkers have reported several amyloid hydrogelators with promising biomedical applications.^38,39^ Not all amyloid peptides cause hydrogelation. Mere self-assembly into fibrillar structures, therefore, does not necessitate hydrogelation. Subtle modifications, however, can dramatically affect the hydrogelation propensity.^40^

Compared to a much better understanding of peptide self-assembly in the past two decades, the understanding of peptide hydrogelation remains primitive. Hydrogelating peptides are often discovered serendipitously or through empirical screening rather than rational design. A deeper understanding of the molecular interactions governing self-assembly, such as hydrogen bonding, dipole–dipole forces, hydrophobic effects, and π–π stacking, should enable the prediction of hydrogelating peptides as well as their emergent properties.^10,41,42^ Aromatic amino acids are crucial in peptide self-assembly, facilitating assembly through π-stacking interactions.^43–47^ The occurrence of phenylalanine and tyrosine is far more than that of tryptophan in self-assembling peptides.^48^ Reches and Gazit reported nanotubes formed by the peptide diphenylalanine.^49^ They subsequently investigated the aromatic homodipeptides wherein halogen groups were attached to the phenyl ring.^44^ Halogen modification had deterministic effects on the assembly of dipeptides and the emergent superstructures. Fmoc-Phe-OH, Fmoc-Tyr-OH, and Fmoc-Phe-Phe-OH have been reported in the literature to form hydrogels.^50–52^ The self-assembly has been attributed to π–π interactions between the aromatic side chains and the Fmoc moiety.

Aβ_16–22_, a well-studied amyloidogenic peptide from β-amyloid, harbors a pair of Phe residues that facilitate self-assembly.^53^ The end-capped Aβ_16–22_ (CH_3_CO-KLVFFAE-NH_2_) self-assembles to form antiparallel β-sheets. We have previously investigated the hydrogelation propensity of end-capped Aβ_16–22_. The peptide fails to form hydrogel up to at least 20 mM concentration.^40^ However, the peptide wherein Phe20 is substituted with Tyr forms hydrogel at as low as 2 mM concentration. A subtle perturbation of the aromatic ring's electronic properties, therefore, can dramatically affect self-assembly and hydrogelation. Nilsson and coworkers investigated the self-assembly and hydrogelation of phenyl-ring substituted Fmoc-Phe-OH. Such modifications alter the molecule's hydrophobicity and electronic properties, thereby influencing π–π interactions and self-assembly.^54–56^ Fmoc-Tyr-OH is a better gelator than Fmoc-Phe. The hydroxyl group is an inductively electron-withdrawing group but an electron-donating group through resonance. The resonance effect usually outweighs the inductive effect of the hydroxyl group. Considering this, the phenolic ring in Fmoc-Tyr-OH is expected to be more electron-rich compared to the phenyl group. In contrast, the inductive effect is stronger than the resonance effect for halogens. Interestingly, Fmoc-F_5_-Phe (Fmoc-protected pentafluorophenylalanine), an amino acid with depleted electron density in the pentafluorophenyl ring, forms better hydrogel than Fmoc-Tyr.^55^ These results indicate that substitutions in the aromatic side chain can dramatically affect self-assembly and hydrogelation. It is important to note that the ring substitutions also contribute to the size of the aromatic side chain and its hydrophobicity. To understand if the aromatic residues confer aggregation propensity through their hydrophobicity and β-sheet propensity, the ring electronic effects, or ring geometry, Desamero and coworkers carried out a detailed investigation on hIAPP_22–29_ (NFGAILSS).^57^ They made many analogs by substituting the Phe23 ring with several electron-withdrawing and electron-donating groups. They found that the peptide analogs with electron-donating groups on the phenyl ring displayed poor aggregation, while those with electron-withdrawing groups displayed better self-assembly. Their results establish that aromatic electronic effects influence peptide self-assembly, with electron-withdrawing substituents promoting it.

We have recently reported tau^306–311^ (CH_3_CO-VQIVYK-NH_2_), an amyloidogenic stretch from human tau, as a promising biocompatible hydrogelator.^58^ Tau is a microtubule-associated protein that gets aggregated in several neurodegenerative diseases, collectively called tauopathies.^59,60^ Mandelkow identified a couple of hexapeptide stretches (tau^306–311^ and tau^275–280^) that act as interaction motifs, facilitating tau self-assembly.^61,62^ Tau^306–311^ self-assembly has been investigated in great detail.^63–68^ CH_3_CO-VQIVYK-NH_2_, known as Ac-PHF6 in the literature, forms a viscous solution in water but causes instant gelation of phosphate-buffered saline and the cell culture media DMEM and RPMI.^58^ The peptide harbors a tyrosine residue. As uncapped VQIVYK is reported in the literature to self-assemble through parallel β-sheet formation where tyrosine residues are involved in aromatic stacking interactions,^69^ we investigated the peptide analogs wherein Tyr was substituted with Phe or ring-substituted Phe (Table 1).

**Table 1:** The sequences of peptides employed in this study

Peptide sequence	Remarks
Ac-VQIVYK-am	Ac-PHF6 (tau^306–311^)
Ac-VQIVFK-am	Tyr → Phe analog
Ac-VQIVF(fl)K-am	Tyr → *p*-fluorophenylalanine analog
Ac-VQIVF(CN)K-am	Tyr → *p*-cyanophenylalanine analog
Ac-VQIVF(NO_2_)K-am	Tyr → *p*-nitrophenylalanine analog
Ac-VQIVF(CF_3_)K-am	Tyr → *p*-(trifluoromethyl)phenylalanine analog

## Materials and methods

### Materials

Rink amide resin, Fmoc-protected natural amino acids, 1-hydroxybenzotriazole hydrate (HOBt), and *N*,*N*,*N*′,*N*′-tetramethyl-*O*-(1*H*-benzotriazol-1-yl)uronium hexafluorophosphate (HBTU) were acquired from Novabiochem. Fmoc-protected non-natural amino acids were purchased from GL Biochem (Shanghai) Ltd. *N*,*N*-Dimethylformamide, *N*,*N*-diisopropylethylamine (DIPEA), trifluoroacetic acid (TFA), acetic anhydride, thioflavin T (ThT), diethyl ether, triisopropylsilane (TIPS), and acetonitrile were procured from Merck.

### Electrostatic charge density mapping

The structure of toluene was downloaded from PubChem, and aromatic moieties were prepared by substituting the *para* hydrogen atom of toluene using Avogadro 1.2 software.^70^ Electrostatic charge density maps of toluene and its substituents were generated by creating the surface in Avogadro. The extended peptide structures (pdb files) were prepared using UCSF Chimera. The electrostatic charge density maps of the peptides in extended conformation were generated using the APBS (Adaptive Poisson-Boltzmann Solver) program in PyMOL.

### Peptide synthesis and characterization

The peptides (listed in Table 1) were assembled on Rink amide resin by employing Fmoc chemistry with HBTU/HOBt/DIPEA activation. N-terminal acetylation was carried out on-resin using 10 equivalents each of acetic anhydride and DIPEA. The peptides were cleaved from the resin using a cleavage cocktail containing 95% TFA, 2.5% TIPS, and 2.5% water. The peptides were precipitated in ice-chilled diethyl ether. Following multiple rounds of washing with diethyl ether, the peptides were air-dried. The peptides were purified using reversed-phase HPLC on a C18 column, employing a linear gradient of acetonitrile with 0.1% TFA. Peptide identities were ascertained using MALDI-TOF mass spectrometry.

### Peptide dissolution, hydrogelation and rheology

Peptide stock solutions were prepared in water by weighing the peptides, adding water, and vortexing for a few minutes. All peptides, except Ac-VQIVF(CF_3_)K-am, dissolved in water to very high concentrations (>20 mM). Ac-VQIVF(CF_3_)K-am was heated for about an hour at 70 °C to achieve a dissolution >20 mM. All the peptides remained as viscous solutions in water up to about 25 mM concentration, except Ac-VQIVF(CF_3_)K-am, which gels upon cooling to room temperature. Gelation of PBS was attempted by diluting the peptide solutions to achieve 20 mM peptide concentration. As Ac-VQIVF(CF_3_)K-am gels upon cooling down to room temperature, it was diluted in PBS instantly after being taken out from 70 °C. All the peptides caused instant gelation of PBS. Rheology of Ac-PHF6 gel is reported in the literature for a 24-hour-old gel.^58^ The 20 mM gel has a storage modulus around 20 kPa from 624 rad s^−1^ down to 0.01 rad s^−1^ frequency. Therefore, we carried out the oscillatory rheology on the 20 mM peptide gels that were aged for 24-hours. Rheology measurements were carried out on an Anton Paar Rheometer MCR 102 using 25 cm parallel plates at a 0.5 mm gap. The amplitude sweep tests were carried out at an angular frequency of 10 rad s^−1^ by varying shear strain from 0.01% to 10%. All the gels showed a linear regime up to at least 0.1% strain. Frequency sweep data, therefore, were recorded at 0.1% strain.

### Thioflavin T (ThT) fluorescence spectroscopy

ThT fluorescence emission spectra were recorded in PBS for the PBS gels. As ThT fluorescence quantum yield strongly depends on pH, the fluorescence emission spectra for water samples were recorded in 10 mM phosphate buffer, pH 7.4, instead of water. The assay was carried out at 200 μM peptide and 10 μM ThT concentrations. The samples were excited at 450 nm, and emission spectra were recorded. The excitation and emission bandwidths were 2.5 and 5 nm, respectively.

### Circular dichroism (CD) spectroscopy

Far-UV electronic CD spectra were recorded on a Jasco J-1500 spectropolarimeter. The 24-hour-old samples (20 mM peptide concentration) were diluted in respective dispersant (water or PBS) to 200 μM concentration, and spectra were recorded in a 1 mm path-length quartz cell. The spectra were recorded from 250–195 nm at 1 nm bandwidth with a scanning speed of 100 nm min^−1^. Each spectrum is the average of 8 accumulations. The spectra were corrected by subtracting the respective dispersant spectrum. The data was converted to mean residue ellipticity using the following formula: 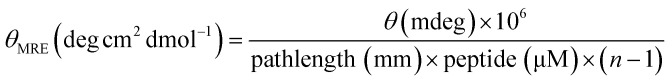, where *n* is the number of amino acids in the peptide.

### Fourier transform infrared (FTIR) spectroscopy

The FTIR spectra were recorded for 24-hour-old samples on a Shimadzu IRAffinity-1S Fourier transform infrared spectrometer equipped with a diamond ATR crystal. The samples were diluted 2-fold, and ∼5 μL volume of the diluted samples was deposited on the diamond crystal and allowed to dry. The spectra were recorded with 4 cm^−1^ resolution and 40 scans.

### Molecular dynamics (MD) simulations

MD simulations were carried out for the steric zipper structures prepared for the peptides. The steric zipper structure of uncapped PHF6 peptide (VQIVYK.pdb) was obtained from the WALTZ-DB database (http://waltzdb.switchlab.org/).^71^ N-terminal acetylation was done in BIOVIA Discovery Studio. The aromatic analogs were generated by substituting the Tyr residue with Phe and ring-substituted-Phe using the SwissSidechain plugin in UCSF-Chimera. MD simulations were carried out using the CHARMM36 force field. The peptide amidation was done by choosing CT2 as the terminal patch when prompted by pdb2gmx command. The steric zippers were placed in a cubic box with a minimum distance of 1 nm from the box edge, solvated with water (TIP3P), and neutralized with chloride ions. NaCl (150 mM) was added to each system. The systems underwent energy minimization, followed by equilibration under NVT (100 ps) and NPT (200 ps) at 300 K temperature and 1 bar pressure. Subsequently, the production MD simulations were carried out for 200 ns. Trajectory analyses were performed using VMD and UCSF-Chimera. Cluster analysis was done using the gromos algorithm with a 0.2 nm cutoff.

### Transmission electron microscopy (TEM)

The gel samples were diluted 2-fold in PBS and deposited on the carbon-coated copper grids. After 10 minutes, the excess solvent was removed using lint-free tissue paper, and the grids were stained with uranyl acetate for 10 minutes before being left to air dry. The images were acquired at 200 kV using a JEM-2100F (JEOL, Japan) transmission electron microscope.

## Results and discussion

Desamero and coworkers investigated hIAPP_22–29_ (NFGAILSS) analogs wherein the phenyl ring was substituted with electron-donating and electron-withdrawing groups.^57^ The peptide analogs with electron-deficient aromatic rings favored self-assembly, while those with electron-donating groups exhibited poor aggregation. Nilsson and coworkers found that incorporating a halogen in the phenyl group of Fmoc-Phe-OH promotes self-assembly and hydrogelation.^54^ Fmoc-F_5_-Phe, an analog with a highly-electron-deficient aromatic ring, displays faster self-assembly and hydrogelation compared to Fmoc-Tyr-OH.^55^ These studies propound that electron-withdrawing moieties on aromatic side chains facilitate peptide self-assembly and hydrogelation. Ac-PHF6 contains a Tyr side chain and causes PBS hydrogelation. Here, we report the hydrogelation of Ac-PHF6 analogs, wherein Tyr310 is replaced with Phe and its ring-substituted derivatives (Table 1).

### Electrostatic charge density map

**Fig. 1:**
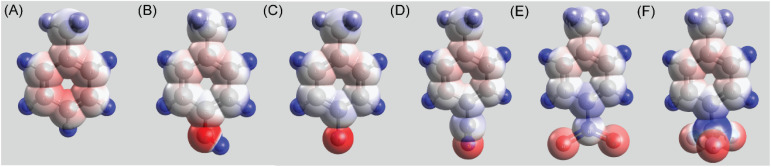
Electrostatic charge density maps of toluene (A), *p*-cresol (B), 4-fluorotoluene (C), 4-cyanotoluene (D), 4-nitrotoluene (E), and 4-(trifluoromethyl)toluene (F).

An electrostatic charge density map provides valuable insights into molecular interactions. It helps in predicting electrostatic interactions that include ionic bonds, hydrogen bonds, and dipolar interactions. The charge density maps for the aromatic side chains incorporated in Ac-PHF6 analogs are shown in Fig. 1. Toluene was used as the model for the Phe side chain, and its substituents were used as models for other aromatic side chains employed in this study. *Para*-cresol, the model for Tyr side chain, shows the highest electron density in the ring (Fig. 1B). All other aromatic groups display lower electron density in the aromatic ring. The surface charge density maps of the peptides listed in Table 1 in their extended conformation are shown in Fig. S1.†

### Hydrogelation

**Fig. 2:**
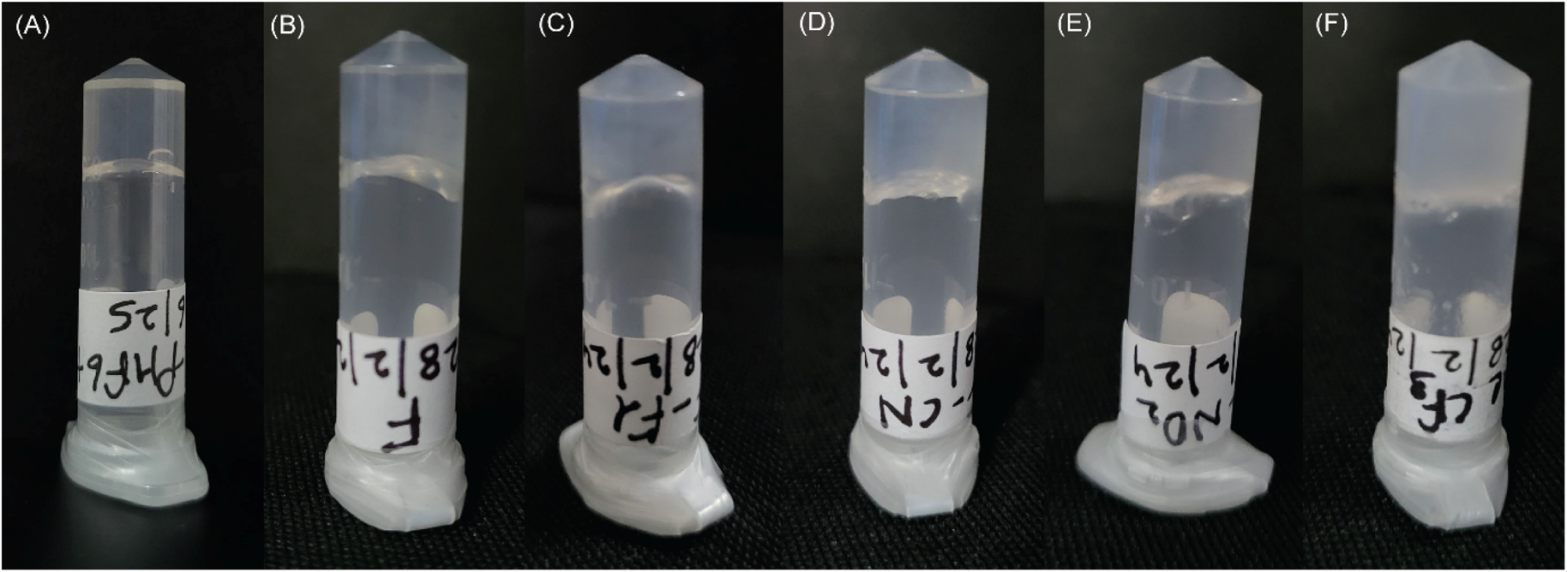
Inverted vials of Ac-PHF6 and its analogs showing PBS gelation. (A) Ac-PHF6, (B) Ac-VQIVFK-am, (C) Ac-VQIVF(fl)K-am, (D) Ac-VQIVF(CN)K-am, (E) Ac-VQIVF(NO_2_)K-am, and (F) Ac-VQIVF(CF_3_)K-am.

Peptide stock solutions were prepared in water. All peptides, except Ac-VQIVF(CF_3_)K-am, readily dissolved in water to >20 mM concentration. Ac-VQIVF(CF_3_)K-am displayed lower solubility and had to be heated at 70 °C for an hour to achieve dissolution. Cooling down to room temperature resulted in hydrogelation. No other peptide caused water gelation. The uncapped PHF6 (VQIVYK) has been reported in the literature to self-assemble in the presence of high salt concentration *via* parallel in-register β-sheet formation.^69^ Capped peptide (Ac-PHF6) is also expected to assemble similarly. The salt masks the intermolecular electrostatic repulsion between terminal Lys residues in parallel β-sheet arrangement. The self-assembly and gelation of Ac-VQIVF(CF_3_)K-am in water is likely hydrophobicity-driven. Trifluoromethyl is a hydrophobic functional group. The high hydrophobicity of *p*-(trifluoromethyl)phenylalanine renders Ac-VQIVF(CF_3_)K-am poorly soluble in water, facilitating its self-assembly through entropic contribution. PBS gelation was set up by diluting the peptide stock solutions in 10× PBS. As Ac-VQIVF(CF_3_)K-am causes water gelation at room temperature, it was diluted immediately after taking out from 70 °C. All the peptides caused PBS gelation. The gels formed at 10 and 15 mM peptide concentrations were very fragile. Firm gels were obtained at 20 mM concentration (Fig. 2). The gelation was instant, but the inverted tube images shown in Fig. 2 were taken for the 24-hour-old samples. As 24-hour-old Ac-PHF6 hydrogel (20 mM peptide concentration) is reported in the literature, the assays with Ac-PHF6 analogs were also carried out with 20 mM gel samples that were incubated at room temperature for 24 hours.

### Rheology

**Fig. 3:**
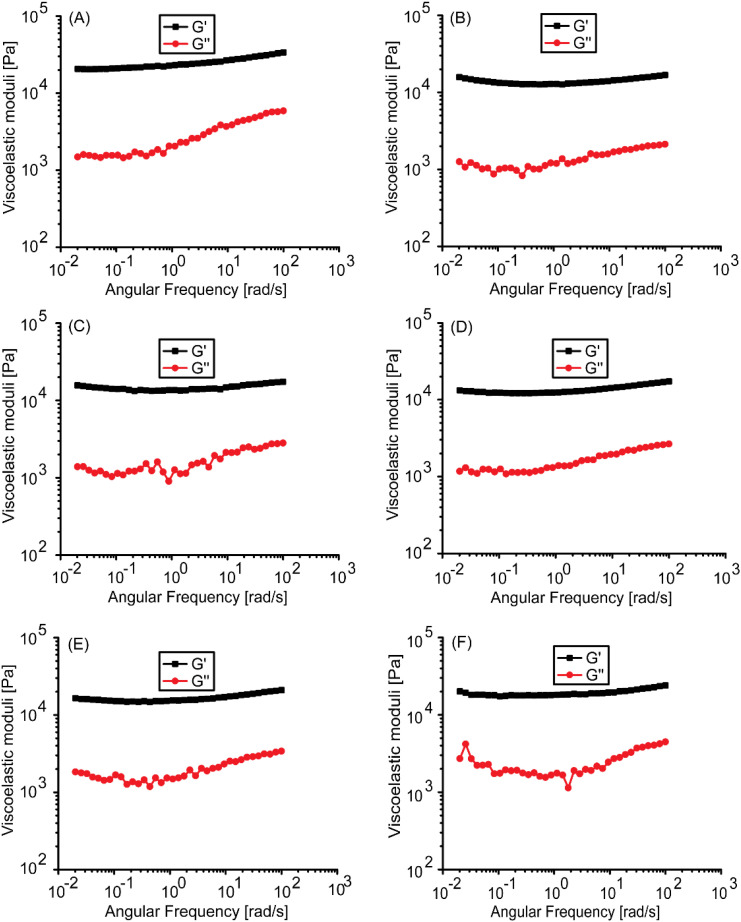
Rheology of PBS gels. Plot of *G*′ and *G*′′ against angular frequency for 20 mM gels of (A) Ac-PHF6, (B) Ac-VQIVFK-am, (C) Ac-VQIVF(fl)K-am, (D) Ac-VQIVF(CN)K-am, (E) Ac-VQIVF(NO_2_)K-am, and (F) Ac-VQIVF(CF_3_)K-am.

Rheology of a material is determined by its inner structure. As aromatic substitutions could modulate the peptide self-assembly, affecting the hydrogel's inner structure, the hydrogels' viscoelasticity was investigated using bulk rheology. The frequency sweep test was conducted at a strain of 0.1%, the strain that lies in the linear viscoelastic regime. The data is shown in Fig. 3. Interestingly, all the gels displayed a comparable storage modulus of about 1 − 2 × 10^4^ Pa. There are small differences in the loss moduli, though. Instant PBS gelation caused by the peptides could lead to some non-uniformity within the gels. Such non-uniformity could contribute to minor differences observed in the loss moduli. The rheology data suggest that Tyr electronic properties do not contribute significantly to Ac-PHF6 hydrogelation. Unlike Fmoc-F_5_-Phe-OH, which forms a much stronger hydrogel than Fmoc-Tyr-OH,^55^ we find that substitution of Tyr in Ac-PHF6 with Phe or its analogs with electron-withdrawing groups has no significant effect on rheology. The only apparent difference to Ac-PHF6 is that Ac-VQIVF(CF_3_)K-am displays lower solubility in water due to the high hydrophobicity of the Phe(CF_3_) group. The higher hydrophobicity caused the peptide to gel in deionized water as well.

### CD spectroscopy

**Fig. 4:**
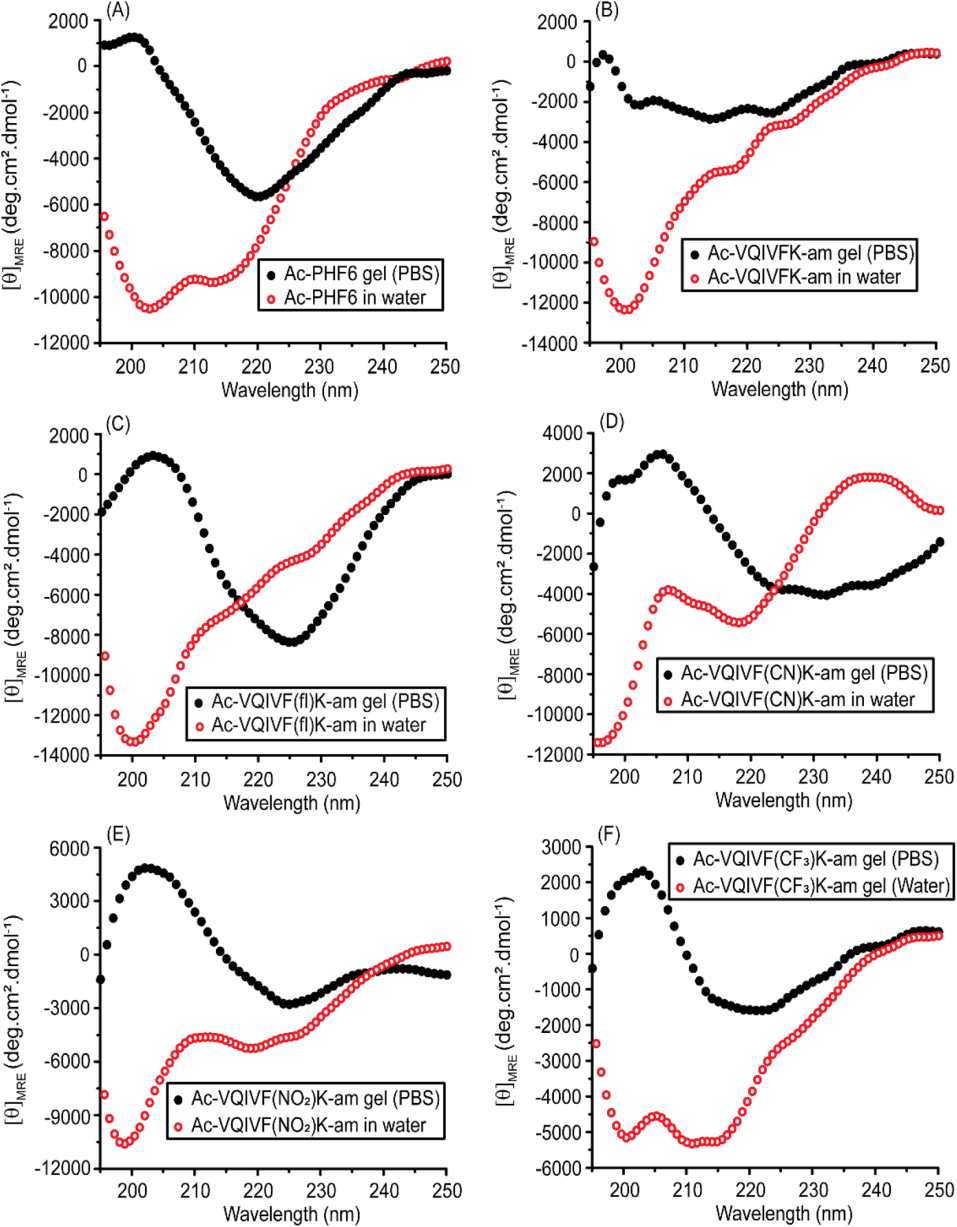
CD spectra of Ac-PHF6 analogs, (A) Ac-PHF6, (B) Ac-VQIVFK-am, (C) Ac-VQIVF(fl)K-am, (D) Ac-VQIVF(CN)K-am, (E) Ac-VQIVF(NO_2_)K-am, and (F) Ac-VQIVF(CF_3_)K-am.

The secondary structures of PBS and water samples were examined using far-UV CD spectroscopy. The samples were diluted to a concentration of 200 μM in the respective dispersant (water/PBS) for CD spectroscopy. Ac-PHF6 displays a spectrum very similar to that reported in the literature (Fig. 4A). The spectrum suggests a largely unordered conformation in water with a little contribution from β-sheets. On the other hand, the peptide displays a distinct β-sheet spectrum in PBS. Ac-VQIVFK-am displays a negative band around 200 nm in water, indicating a largely unordered conformation (Fig. 4B). The peptide displays a broad band centered around 215 nm in PBS, suggesting a β-sheet conformation. Ac-VQIVF(fl)K-am also takes up a largely unordered conformation in water, as indicated by the 200 nm negative band (Fig. 4C). The spectrum in PBS is characterized by a broad band around 225 nm and a weak positive band around 205 nm. The spectrum is very similar to that reported for Ac-PHF6 in PBS and is assigned to the β-sheet conformation. Ac-VQIVF(CN)K-am (Fig. 4D) and Ac-VQIVF(NO_2_)K-am (Fig. 4E) display a negative band around 220 nm alongside the band around 200 nm in water. These data indicate that a fraction of the peptide takes up the β-sheet conformation, suggesting a tendency to self-assemble in water. In PBS, both the peptides display distinct β-sheet conformation. Ac-VQIVF(CF_3_)K-am displays a very different spectrum in water (Fig. 4F). The spectrum is characterized by 200 and ∼212 nm negative bands of comparable amplitudes. The spectrum suggests that the peptide takes up a mixture of β-sheet and random coil conformations. In PBS, a typical β-sheet CD spectrum is observed. The CD data show that the peptides with strong electron-withdrawing groups *i.e.*, cyano, nitro, and trifluoromethyl groups, have β-sheet content in water, suggesting their tendency to self-assemble in water itself. Ac-VQIVFK-am and Ac-VQIVF(fl)K-am, *i.e.*, the peptides without an electron-withdrawing group and with a weak electron-withdrawing group, respectively, show largely unordered conformation. The most dramatic effect of the electron-withdrawing group is observed for Ac-VQIVF(CF_3_)K-am, which displays lower solubility in deionized water at room temperature and causes gelation upon cooling after being heated.

### FTIR spectroscopy

**Fig. 5:**
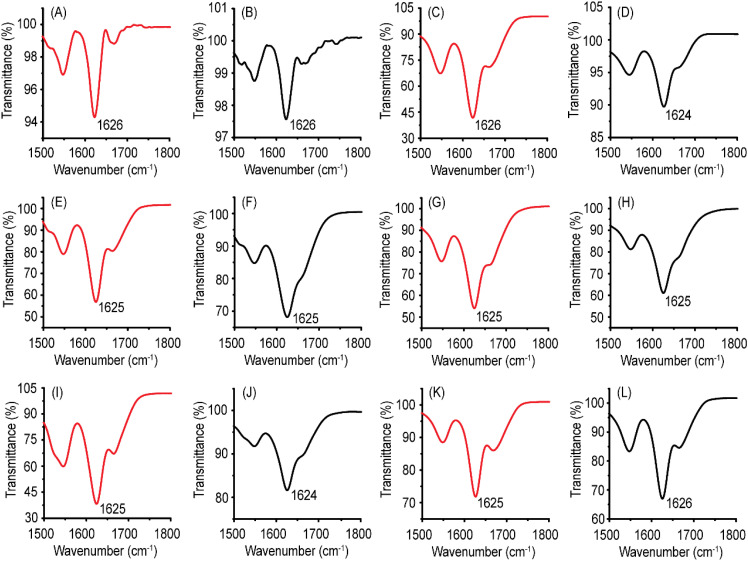
ATR-FTIR spectra of Ac-PHF6 and its analogs. The red traces are the spectra recorded for water samples, while the black traces are the spectra recorded for PBS samples. (A and B) Ac-PHF6, (C and D) Ac-VQIVFK-am, (E and F) Ac-VQIVF(fl)K-am, (G and H) Ac-VQIVF(CN)K-am, (I and J) Ac-VQIVF(NO_2_)K-am, and (K and L) Ac-VQIVF(CF_3_)K-am.

CD spectroscopy is an excellent method for investigating the α-helical conformations. However, the huge structural diversity of β-sheets makes CD spectroscopy somewhat less reliable for accurately estimating the β-sheet conformation.^72^ Regarding self-assembling peptides with aromatic residues, contributions from aromatic stacking interactions in far-UV CD spectra further hinder secondary structure prediction. FTIR spectroscopy is particularly suitable for investigating the β-sheets. The position of the amide I band is sensitive to the peptide backbone conformation. The ATR-FTIR spectra of Ac-PHF6 and its analogs are shown in Fig. 5. All the peptides display the amide I band centered between 1624–1626 cm^−1^ for both water and PBS samples. ATR-FTIR data unambiguously proves that the peptides take a β-sheet conformation in the hydrogels. Unlike CD spectroscopy, where Ac-VQIVFK-am and Ac-VQIVF(fl)K-am display random coil conformation for water samples, the FTIR spectra indicate β-sheet conformation. This is attributed to the drying of the peptides for ATR-FTIR spectroscopy. The peptide concentration increases during the drying processes, facilitating self-assembly. Such behavior has been reported for Ac-PHF6 in the literature.^58^

### ThT fluorescence

**Fig. 6:**
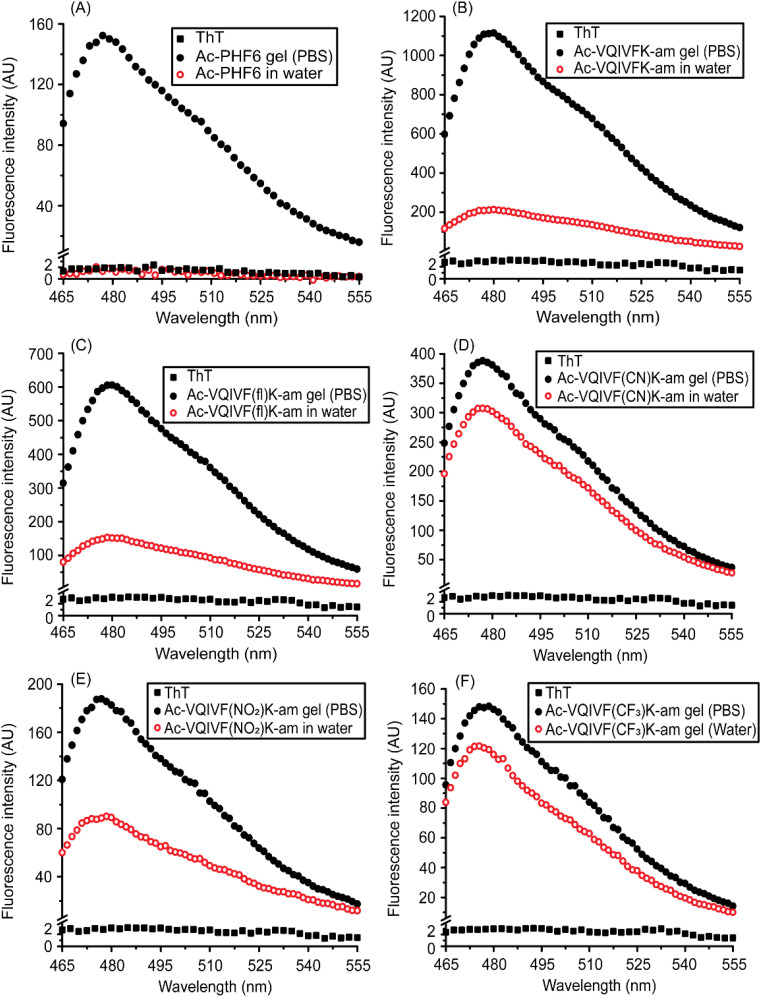
ThT fluorescence spectra of Ac-PHF6 and its analogs. (A) Ac-PHF6, (B) Ac-VQIVFK-am, (C) Ac-VQIVF(fl)K-am, (D) Ac-VQIVF(CN)K-am, (E) Ac-VQIVF(NO_2_)K-am, and (F) Ac-VQIVF(CF_3_)K-am.

The fluorescence of ThT, a benzothiazole dye that demonstrates a higher quantum yield when bound to amyloid fibrils, is routinely used to characterize amyloid-like fibrils. ThT fluorescence spectra are shown in Fig. 6. Ac-PHF6 gel sample caused enhancement in ThT fluorescence emission intensity, while no noticeable enhancement was observed for the water sample (Fig. 6A). This data is similar to that reported in the literature.^58^ All the peptides caused a large enhancement in ThT fluorescence intensity, confirming that the superstructures underlying the hydrogels are amyloid-like fibrils. The PBS samples caused a larger enhancement in ThT fluorescence than the water samples. Ac-VQIVFK-am and Ac-VQIVF(fl)K-am display around 4–6 times higher intensity for PBS samples compared to water samples (Fig. 6B and C). Interestingly, however, the ThT fluorescence observed for Ac-VQIVF(NO_2_)K-am PBS is about 2-fold higher than the water sample (Fig. 6E). This difference in intensity further decreases for Ac-VQIVF(CN)K-am and Ac-VQIVF(CF_3_)K-am samples (Fig. 6D and F). The PBS samples of these peptides display only about 25% higher ThT fluorescence intensity than the water samples. These data align with the CD spectroscopy data and suggest that the Ac-PHF6 analogs wherein the aromatic residue has a strong electron-withdrawing group, have a higher aggregation propensity.

### MD simulations

**Fig. 7:**
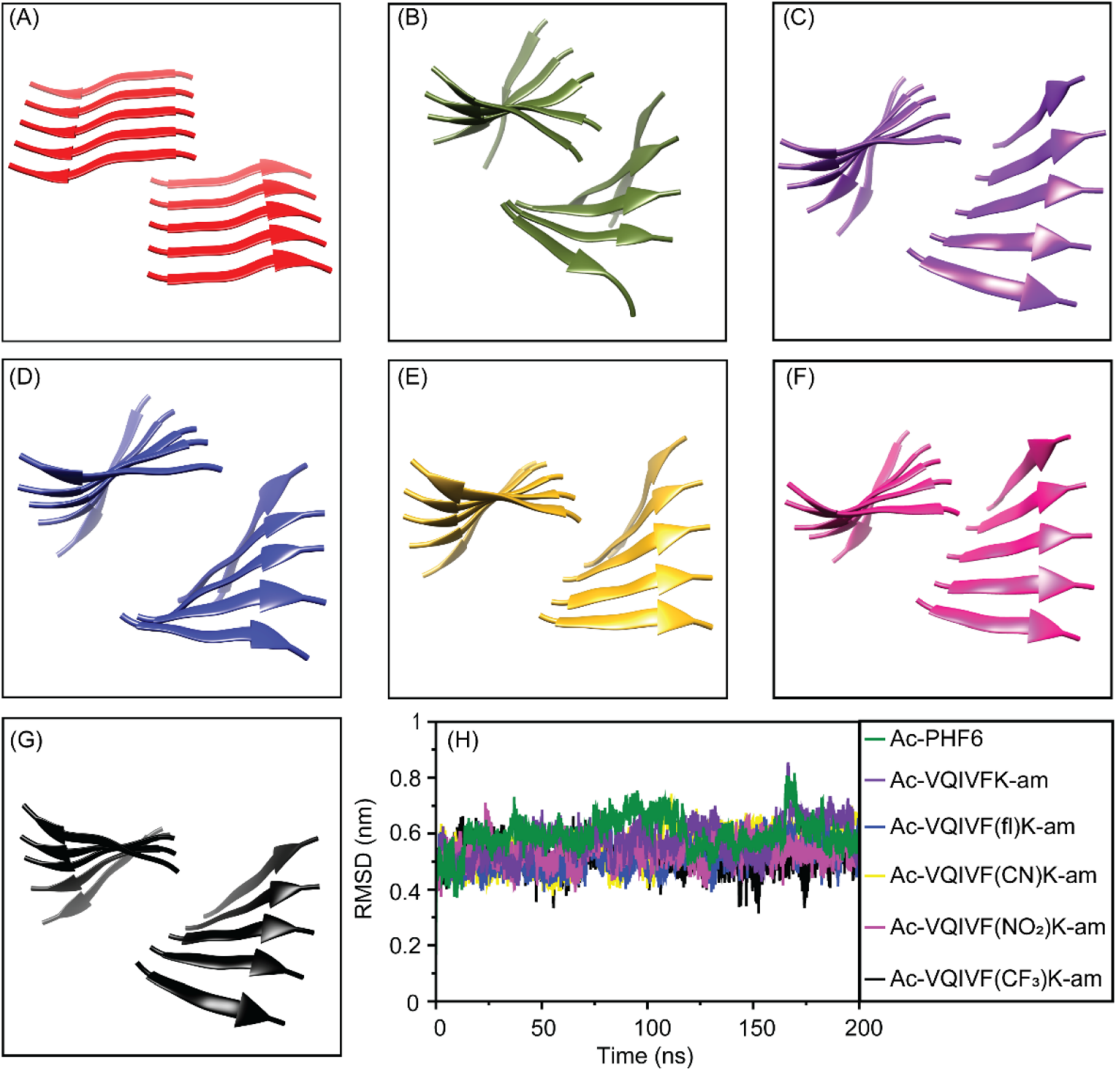
MD simulations of the peptide steric zippers. (A) The VQIVYK steric zipper obtained from WALTZ-DB database. The middle structure of the largest cluster for (B) Ac-PHF6, (C) Ac-VQIVFK-am, (D) Ac-VQIVF(fl)K-am, (E) Ac-VQIVF(CN)K-am, (F) Ac-VQIVF(NO_2_)K-am, and (G) Ac-VQIVF(CF_3_)K-am. (H) The RMSD plots obtained from the trajectories of simulations.

As all Ac-PHF6 analogs formed hydrogels in PBS with very similar rheological properties, we got curious to investigate the stability of the steric zippers formed by them using MD simulations. The starting structures of the peptides were prepared by modifying the VQIVYK.pdb steric zipper structure (Fig. 7A) available in WALTZ-DB database. The simulation data was subjected to cluster analysis to find the most representative structure of the largest cluster. The middle structures of the largest cluster are shown in Fig. 7B–G. The largest cluster shows steric zipper arrangement for all six peptides. The RMSD plots (Fig. 7H) also show no appreciable deviation throughout the 200 ns simulation. These data suggest that parallel in-register β-sheet steric zipper is a stable architecture for Ac-PHF6 and its analogs.

### Transmission electron microscopy

**Fig. 8:**
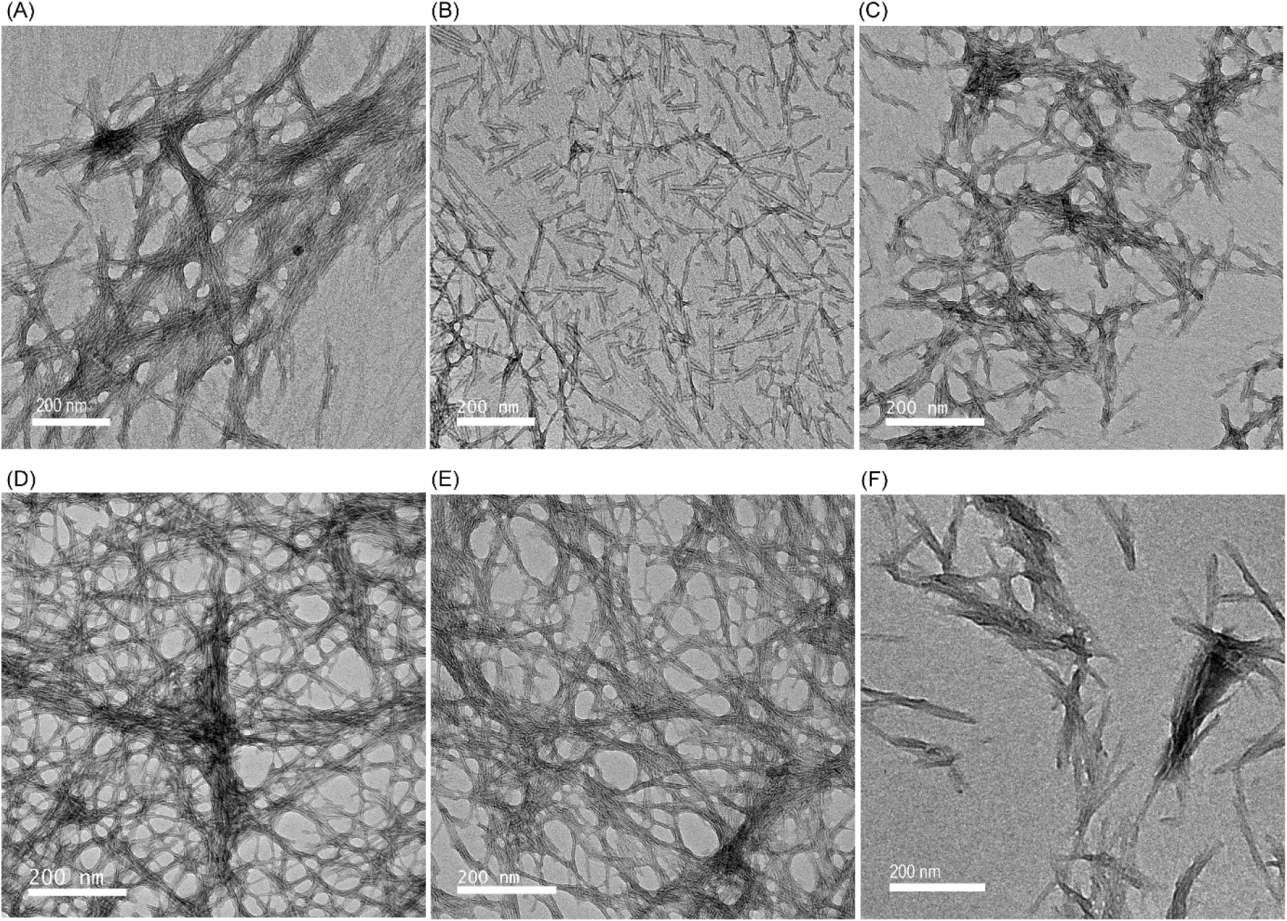
TEM images showing the structures underlying the PBS gels. (A) Ac-PHF6, (B) Ac-VQIVFK-am, (C) Ac-VQIVF(fl)K-am, (D) Ac-VQIVF(CN)K-am, (E) Ac-VQIVF(NO_2_)K-am, and (F) Ac-VQIVF(CF_3_)K-am. The scale bars represent 200 nm.

The comparable viscoelastic properties observed for all peptides indicate that the inner structure underlying the hydrogels could also be similar. The morphology of the self-assembled structures underlying the hydrogels, therefore, was investigated using TEM. All peptides self-assemble into fibrillar superstructures. Ac-PHF6 formed fibrils similar to those reported in the literature (Fig. 8A).^58,63^ Ac-VQIVFK-am and VQIVF(fl)K-am also self-assemble to form straight fibrils (Fig. 8B and C), similar to Ac-PHF6.^58,63^ Ac-VQIVF(CN)K-am and Ac-VQIVF(NO_2_)K-am form long straight filaments that show extensive entanglement (Fig. 8D and E). Ac-VQIVF(CF_3_)K-am forms fibrils that appear to form clumps, possibly through lateral interactions between individual fibrils (Fig. 8F).

## Conclusions

Ac-PHF6, an amyloidogenic stretch from human tau, is a promising biocompatible hydrogelator. As the peptide harbors a Tyr residue, and analogs with electron-withdrawing groups in the aromatic rings of self-assembling peptides have been reported in the literature to form stronger gels, we investigated aromatic analogs of Ac-PHF6. The Ac-PHF6 analogs with electron-withdrawing groups in the phenyl ring improve the peptide's self-assembling propensity. The analog with the strongest electron-withdrawing group (trifluoromethyl), *i.e.*, Ac-VQIVF(CF_3_)K-am causes gelation of deionized water, an attribute that all other peptide analogs lacked. Notably, among all aromatic amino acids employed in this study, the *p*-(trifluoromethyl)phenylalanine has the highest hydrophobicity. The aromatic amino acids incorporated in Ac-PHF6 analogs have very different electronic and steric properties, but all the peptides formed PBS gels with comparable stiffness. The aromatic moieties' contribution to hydrogelation appears more through their hydrophobicity than the aromatic electronic effects. The role of Tyr residue in Ac-PHF6, therefore, is that of a bulky hydrophobic residue rather than an aromatic one. It would be interesting to investigate if the aliphatic analogs of Ac-PHF6 also cause hydrogelation.

## Author contributions

Shubhangini Singh Verma: methodology, validation, formal analysis, investigation, writing – original draft, visualization. Nitin Chaudhary: conceptualization, resources, writing – review & editing, supervision, formal analysis, project administration, funding acquisition.

## Conflicts of interest

There are no conflicts of interest to declare.

## Supplementary Material

RA-015-D5RA03251B-s001

## Data Availability

All data generated in this study are available in the article and the ESI.†
