# Supplementary material for: Substitution of tyrosine with electron-deficient aromatic amino acids improves Ac-PHF6 self-assembly and hydrogelation
Source: RSC Adv. 2025 Jun 30;15(28):22216–27. doi: 10.1039/d5ra03251b (PMC12208052; doi:10.1039/d5ra03251b)
Supplement: RA-015-D5RA03251B-s001 [file RA-015-D5RA03251B-s001.pdf]

Supplementary Information (SI) for RSC Advances.
This journal is © The Royal Society of Chemistry 2025

SUPPLEMENTARY INFORMATION

Substitution of tyrosine with electron-deficient aromatic amino acids improves Ac-PHF6 self-assembly and hydrogelation

Shubhangini Singh Verma and Nitin Chaudhary*

Department of Biosciences and Bioengineering, Indian Institute of Technology Guwahati, Guwahati – 781 039, India

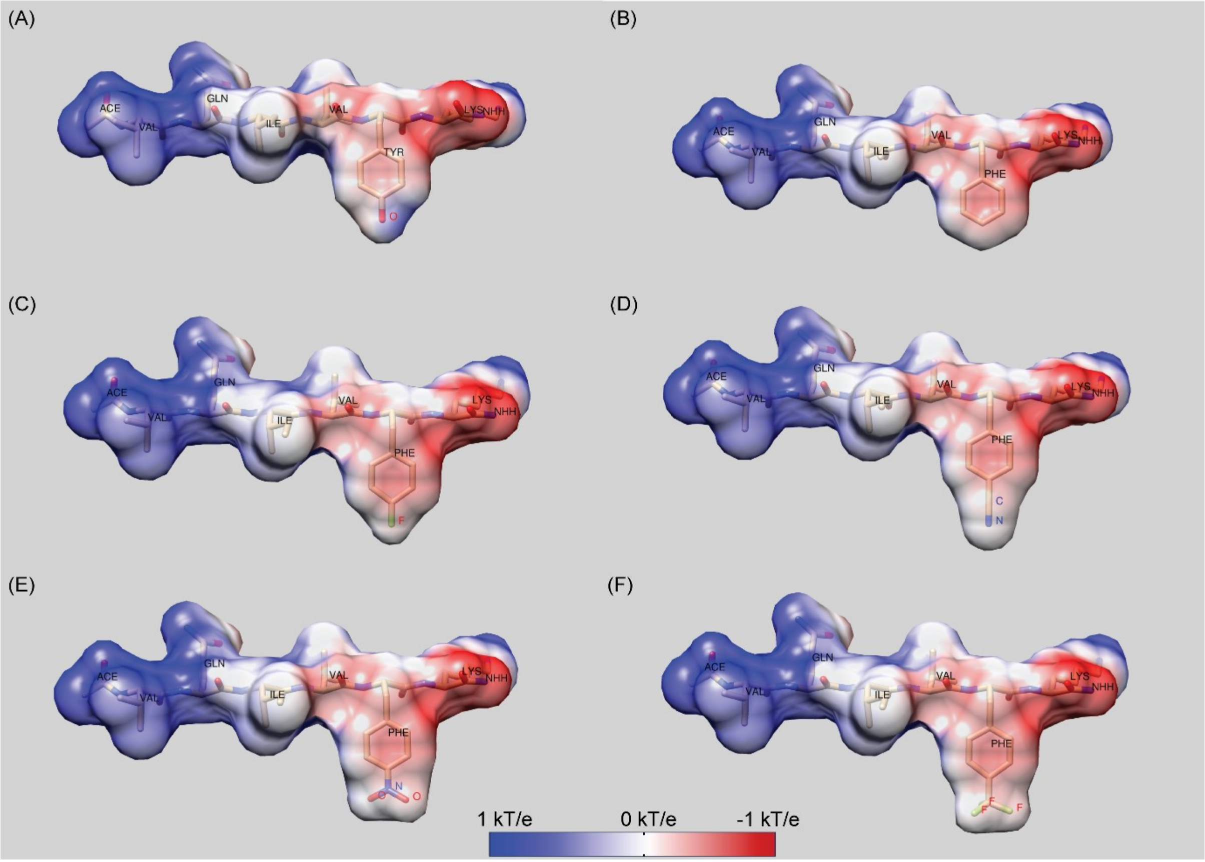

Fig. S1. Electrostatic surface charge density maps of (A) Ac-PHF6, (B) Ac-VQIVFK-am, (C) Ac-VQIVF(fl)K-am, (D) Ac-VQIVF(CN)K-am, (E) Ac-VQIVF(NO_2)K-am, and (F) Ac-VQIVF(CF_3)K-am.

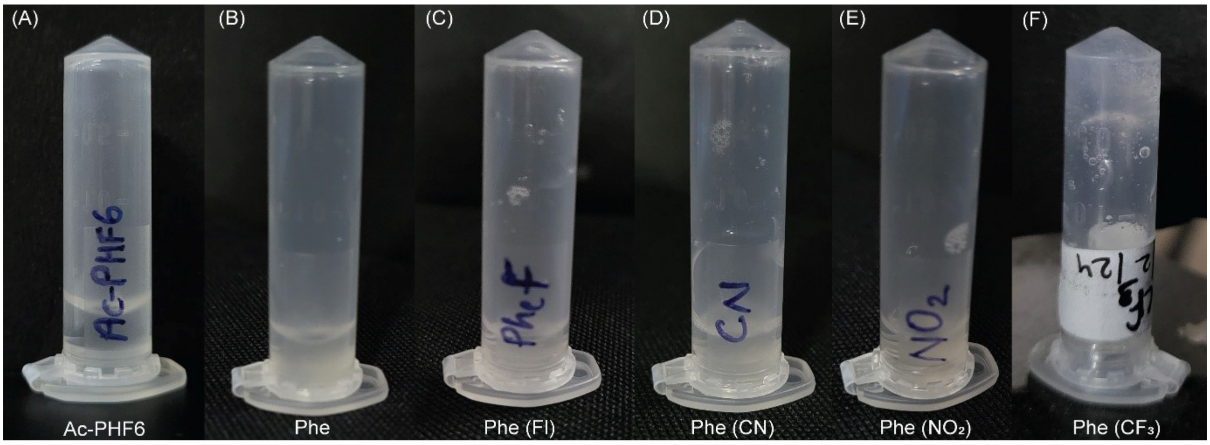

Fig. S2. Inverted vials containing >20 mM stock solutions of Ac-PHF6 and its analogs in deionized water. (A) Ac-PHF6, (B) Ac-VQIVFK-am, (C) Ac-VQIVF(fl)K-am, (D) Ac-VQIVF(CN)K-am, (E) Ac-VQIVF(NO_2)K-am, and (F) Ac-VQIVF(CF_3)K-am.
